# Atlanta Mineral Spring

**Published:** 1859-10

**Authors:** A. Means


### ATLANTA MINERAL SPRING.

We are much gratified in being allowed to present our readers the analysis of the waters of Atlanta Mineral Spring, completed September 16th, 1859, by the celebrated Chemist, Dr. A. Means. Next week we will give an account of the medical properties of these waters, and the diseases to which they are applicable. We return our thanks to Dr. Means, who has so kindly furnished the Analysis:

*Estimates made upon One Gallon, Imperial Measure.*

Specific Gravity, (distilled water being 1,)............1.6005
Temperuture..........................................66 Far.
Quantity per hour.....................................32⅛ gal.

*Gaseous Contents.*

Carbonic Acid...................................7.95 cub. inch.
Hydro-Sulphuric Acid..........................2.33 "
Atmospheric Air, about.......................... 1½ per cent.

*Solid Contents.*

Iron, as a Proto-Carbonate, suspended in Carb.
Acid Gas..........................................14.34 grs.
Sulphate of Magnesia..................................11.74 "
Carbonate of Magnesia.............................. 4.15 "
Magnesia, as base in both.......6.01
Sulphate of Soda.................................... 8.82 "
Chloride of Sodium..................................16.06 "
Lime— .............................................a trace.
Silica—not estimated.
Entire solid contents......... ..........................55.11 grs.

The above combinations, it will be seen, consist mainly of Sulphuric and Carbonic Acids, and Chlorine, with Iron, Magnesia, and Sodium, and are estimated upon Dr. Murray's plan—the several electro-negative portions of the compound being regarded as dividing these electro-positive bases, in accordance with the strength of their several affinities, and the respective solubility of the salts formed.

A. MEANS.

(*Med. & Lit. Weekly.*

